# Comparative Life Cycle Assessment of Cellulose Nanofibres Production Routes from Virgin and Recycled Raw Materials

**DOI:** 10.3390/molecules26092558

**Published:** 2021-04-28

**Authors:** Paola Gallo Stampino, Laura Riva, Carlo Punta, Graziano Elegir, Daniele Bussini, Giovanni Dotelli

**Affiliations:** 1 Politecnico di Milano, Department of Chemistry, Materials and Chemical Engineering “G.Natta”, p.zza L. da Vinci 32, 20133 Milano, Italy; carlo.punta@polimi.it (C.P.); giovanni.dotelli@polimi.it (G.D.); 2 Innovhub Stazioni Sperimentali per l’Industria S.r.l., Via Giuseppe Colombo 83, 20133 Milano, Italy; graziano.elegir@mi.camcom.it (G.E.); daniele.bussini@mi.camcom.it (D.B.)

**Keywords:** nanocellulose, cellulose nanofibres, LCA, TEMPO-mediated oxidation, ENZHO, TOHO, TOSO

## Abstract

Nanocellulose-based materials are attracting an increasing interest for the positive role they could play in sustainable development; being originated from renewable resources. Moreover, cellulose has a high potential of recycling from both post-consumer waste and industrial waste. Both factors, i.e., recyclability and renewable resources; results are also extremely favourable in the perspective of circular economy. Despite all these positive aspects, an industrial production has yet to start. At the lab scale, many preparation methods of cellulose nanofibres (CNF) are available; here, the three most common are analysed: (1) enzymatic pre-treatment followed by homogenisation (ENZHO), (2) oxidative pre-treatment combined with homogenisation (TOHO) or (3) oxidative pre-treatment followed by sonication (TOSO). All three processes have been experimentally carried out starting from both virgin and recycled cellulose from industrial waste sludge. The environmental sustainability of these three routes is estimated by the Life Cycle Assessment (LCA) using experimental lab scale data. In this scenario, the comparative LCA has pointed out a superior performance of the ENZHO process, followed by TOHO and, lastly, by TOSO. The influence of energy consumption on the final results has been further investigated by a sensitivity analysis, showing that the TOHO and TOSO routes could reach similar performances by scaling-up the process from the laboratory. The different typology of CNF obtained by conducting the ENZHO process with respect to the TEMPO-mediated oxidation approach is also outlined as an additional element to be considered for the final selection of a suitable process.

## 1. Introduction

Nowadays, the continuous demand for technology innovation in material designs is facing more and more with the increasing concerns due to waste production and disposal and, more in general, to the impact that the production process would have on the environment. 

An ideal route to minimise this effect would be to follow the principles of circular economy, namely to manage waste as a source for developing up-scaled products. This approach would earn even much more value in terms of sustainability if the disposed waste is derived from a bio-based and renewable source, in order to guarantee since the very beginning high safety standards [1,2].

Cellulose is the most abundant biopolymer on Earth. Formed by the repetition of a high number of β-d-glucopyranose monomers linked together by β-1,4 glycosidic bonds, cellulose shows a high molecular weight and a hierarchical structure, where single fibres are made of microfibrils, which, in turn, are constituted by nanofibres. Cellulose is biodegradable and biocompatible and can be extracted by a wide range of renewable sources, including agricultural waste and recycled paper pulp. In this latter case, however, fibres do not exhibit the desired length and strength for standard uses, which could limit the valorisation of this cellulosic biomass [3].

An attractive route to reach the goal of producing high added-value materials starting from these bio-based waste sources is to cleave the hierarchical structure of cellulose, converting macro-fibrils into nanocellulose (NC) [4,5,6]. Due to the reduction of the size of at least one dimension under 100 nm, NC shows new properties compared to the bulk source, including thermal resistance, mechanical performance, and high surface area and reactivity, so that it is possible to introduce on the nano-skeleton different functional groups. For this reason, it has found ample applications in different fields, including packaging [7], construction materials [8,9], and environmental remediation [10,11,12,13].

NC can be obtained in the form of cellulose nanocrystals (CNC) and cellulose nanofibres (CNF) [6]. The latter are characterised by both amorphous and crystalline domains, with a diameter of 10–100 nm and a length up to several micrometres, depending on the chemical and/or mechanical technique used for this production.

For a long time, CNF have found great applications at the laboratory level as additives or building blocks for different products but poor attention at the industrial level, due to the energy-intensive processes required for their production [14]. Nevertheless, in the last decade, the interest around these nanofibres has progressively increased, due to the introduction of new, much more environmentally and economically sustainable protocols. Nowadays, many companies produce CNF in the order of hundreds of kilograms per day for several applications [15].

**Scheme 1:**
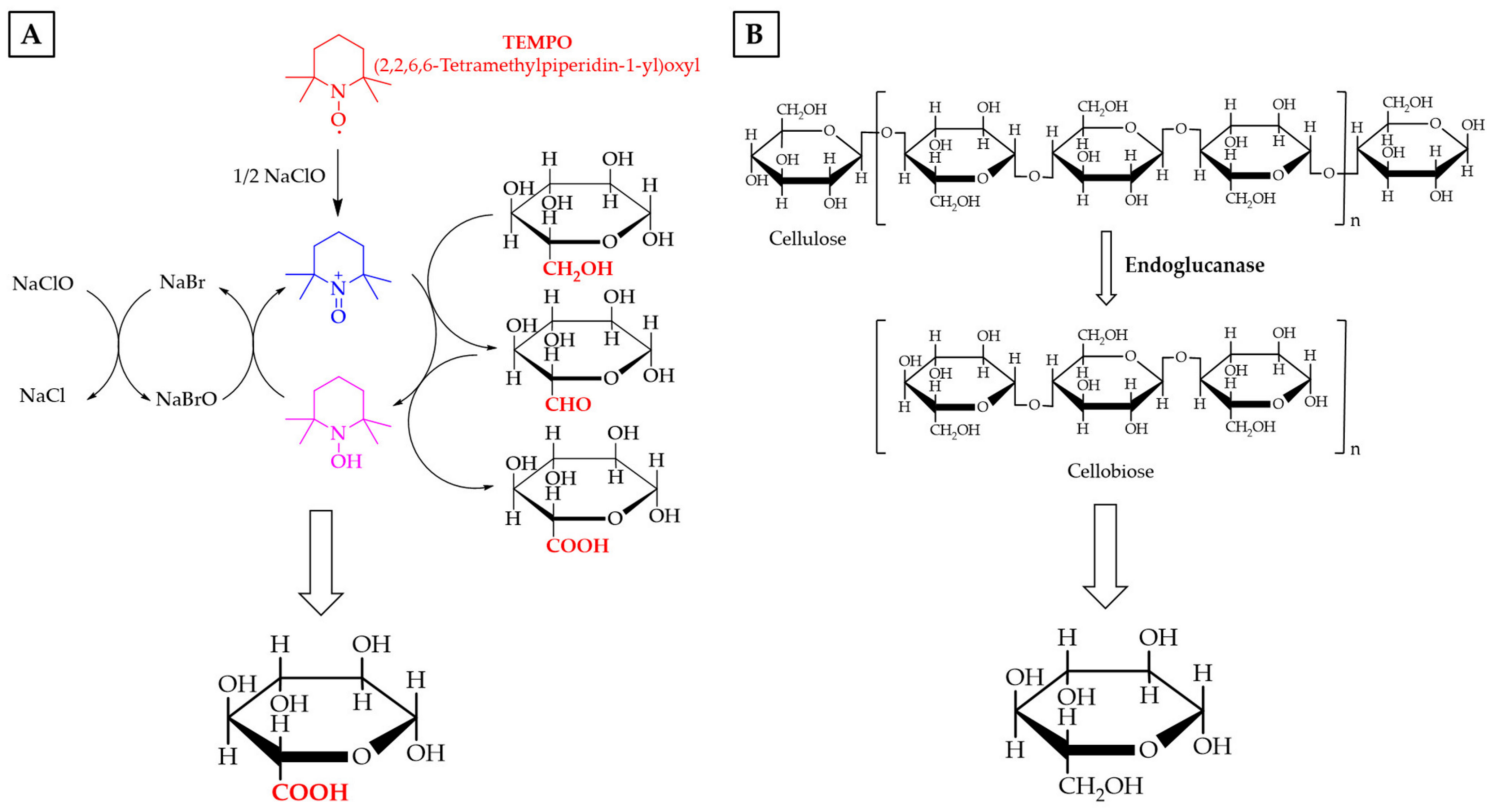
Chemical structure of celluloses obtained by oxidative pre-treatment (**A**) and enzymatic pre-treatment (**B**).

Among the new up–down processes proposed in the last few years for producing CNF, 2,2,6,6-tetramethylpiperidine 1-oxyl (TEMPO)–mediated oxidation (Scheme 1A) and the enzymatic (endoglucanase) pre-treatment (Scheme 1B) represent the most promising ones for sustainable industrial scale-up.

The first approach consists of converting the C6 alcoholic groups of the glucopyranosic units into the corresponding carboxylic moieties by means of the TEMPO/NaClO/KBr oxidative system [16,17]. This chemical transformation allows promoting CNF formation at basic pH by taking advantage of the negative charges introduced on cellulose skeletons as a consequence of the deprotonation of the carboxylic acids. The electrostatic repulsion among the single nanofibres, combined with proper ultrasonication or homogenisation mechanical actions, leads to the production of pre-functionalised CNF.

The enzymatic protocol consists of the selective hydrolysis of specific components in cellulose fibres [18]. In particular, endoglucanase is suggested as a valuable cellulose pre-treatment in place of the standard mineral acid for the disintegration of fibre pulp. Even if, in this case, yields are much lower than those obtained by classic sulfuric acid treatments, the clean chemical process potentially meets the demand for a sustainable production [19,20].

A key step to determining the most sustainable nanocellulose production process and the environmental impact of the different approaches consists of their detailed Life Cycle Assessment (LCA), not only to compare the different methodologies but, also, to drive improvement actions.

### LCA of Nanocellulose: State-of-the-Art

The Life Cycle Assessment is a quantitative methodology used to estimate the relative potential environmental aspects of products, processes, and services, as clearly stated in the standards that are regulating it [21,22]. Now, the LCA has reached a high level of maturity and is accepted worldwide as one of the most effective methods to assess the environmental sustainability [23,24] within the framework of the three-pillars theory of sustainability.

Despite the high interest raised by nanocellulose materials [25] for their possible role in sustainable development [26], few works have addressed the environmental aspects of their production using the LCA methodology [21,22] in the last decade. Indeed, a recent review that appeared in 2021 [27] listed less than 10 works focused on the LCA of nanocellulose materials [28,29,30,31,32,33,34,35]. The review gives a complete account of the main features of the LCA methodology as applied, and for this reason, these works are here not further discussed. Another review has recently focused on the LCA of the fabrication of a larger class of nanobiopolymers, including nanocellulose; the environmental aspects of nanocellulose extraction are discussed in a more general perspective but less focused on LCA technicalities [36].

Other studies that have not been included in these two reviews deserve mention, because they applied the LCA methodology to novel materials obtained from nanocellulose or developed hybrid approaches to support a decision policy. Bartolozzi et al. evaluated the production of nanosponges from nanocellulose in the cradle-to-gate scenario. Three production systems were first investigated at the lab level and then analysed in a scale-up system [37]. Another work investigated a novel nanomaterial, i.e., cellulose nanocrystal foam, by integrating the ex-ante LCA with an environmental health and safety (EHS) screening [38]. One study applied a hybrid approach, i.e., an integrated analytic hierarchy process incorporating LCA, to identify the best production alternative among three different preparation routes of nanocrystalline cellulose extracted from an oil palm empty fruit bunch [39]. 

A recent publication has investigated the role of different impact assessment methods in the final LCA results [40]. In this work, the authors analysed the LCA from cradle-to-the-grave of nanofibrillated cellulose from thermo-groundwood. Nanocellulose fibres were isolated by a chemo–mechanical method using a high-pressure homogenisation technique; in particular, the two last stages of the preparation route were TEMPO oxidation and homogenisation (hereafter, shortly referred to as TOHO). Another work analysed the enzymatic route in great detail from the experimental point of view but included only two indicators in the LCA study [20].

**Figure 1:**
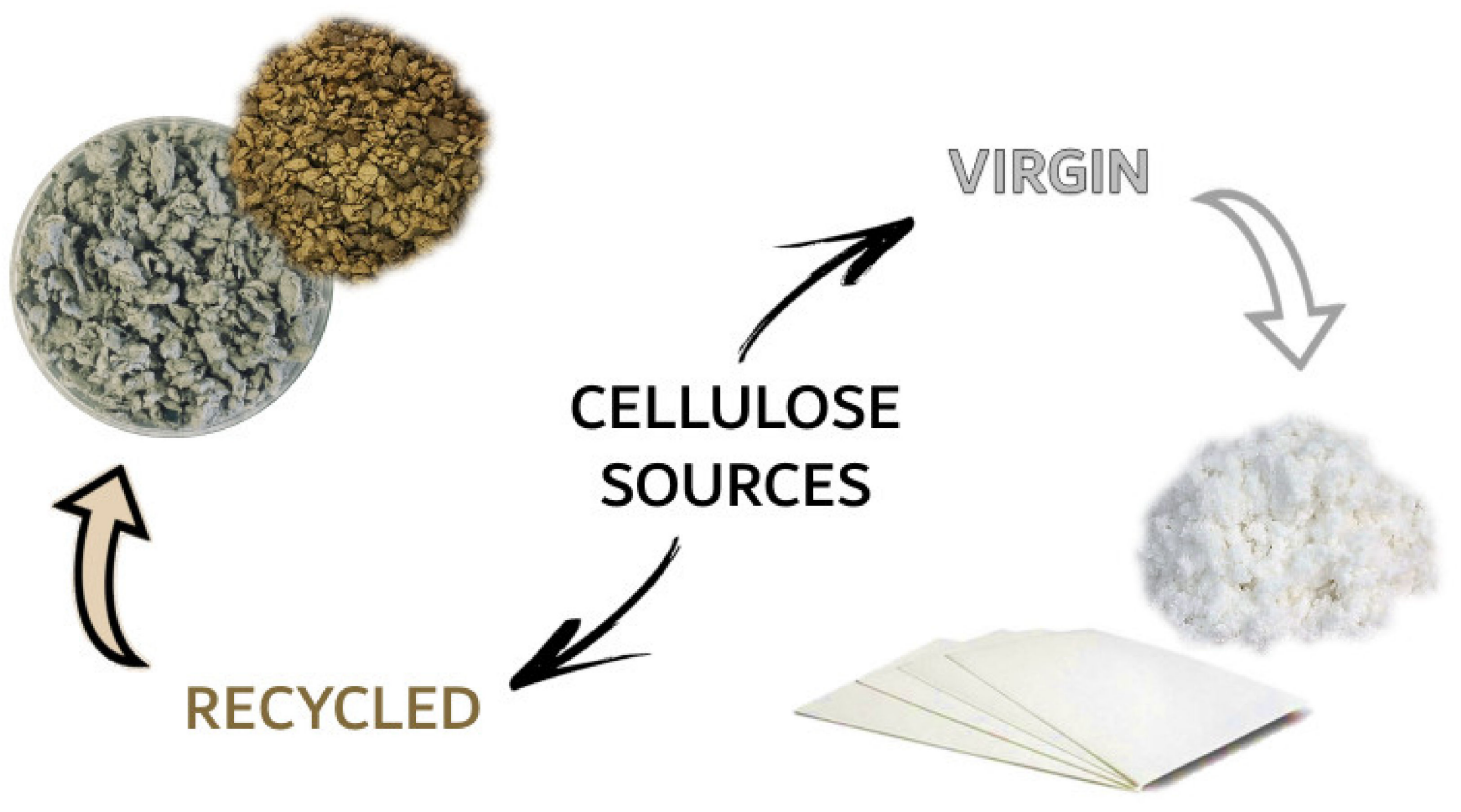
Cellulose sources considered for the production of cellulose nanofibers.

In the present paper, a systematic analysis of the most promising synthetic routes for CNF production, i.e., TEMPO oxidation and enzymatic treatment, are investigated at the lab scale, combining both experimental characterisation of CNFs and environmental impact assessment via LCA methodology. The analysis was conducted on both virgin and waste sources (Figure 1). This work has the ambition to start filling in the existing gap between laboratory and industrial scale-ups by fostering the transition toward an industrial production of CNFs. In the recent literature, we could not find any similar comparative analysis [27], although interesting works have just appeared [40]; last, but not least, the present work strongly relies on laboratory experiments that have fed a consistent and robust set of primary data to the LCA study.

## 2. Results and Discussion

### 2.1. Cellulose Nanofibre Characterisation

**Figure 2:**
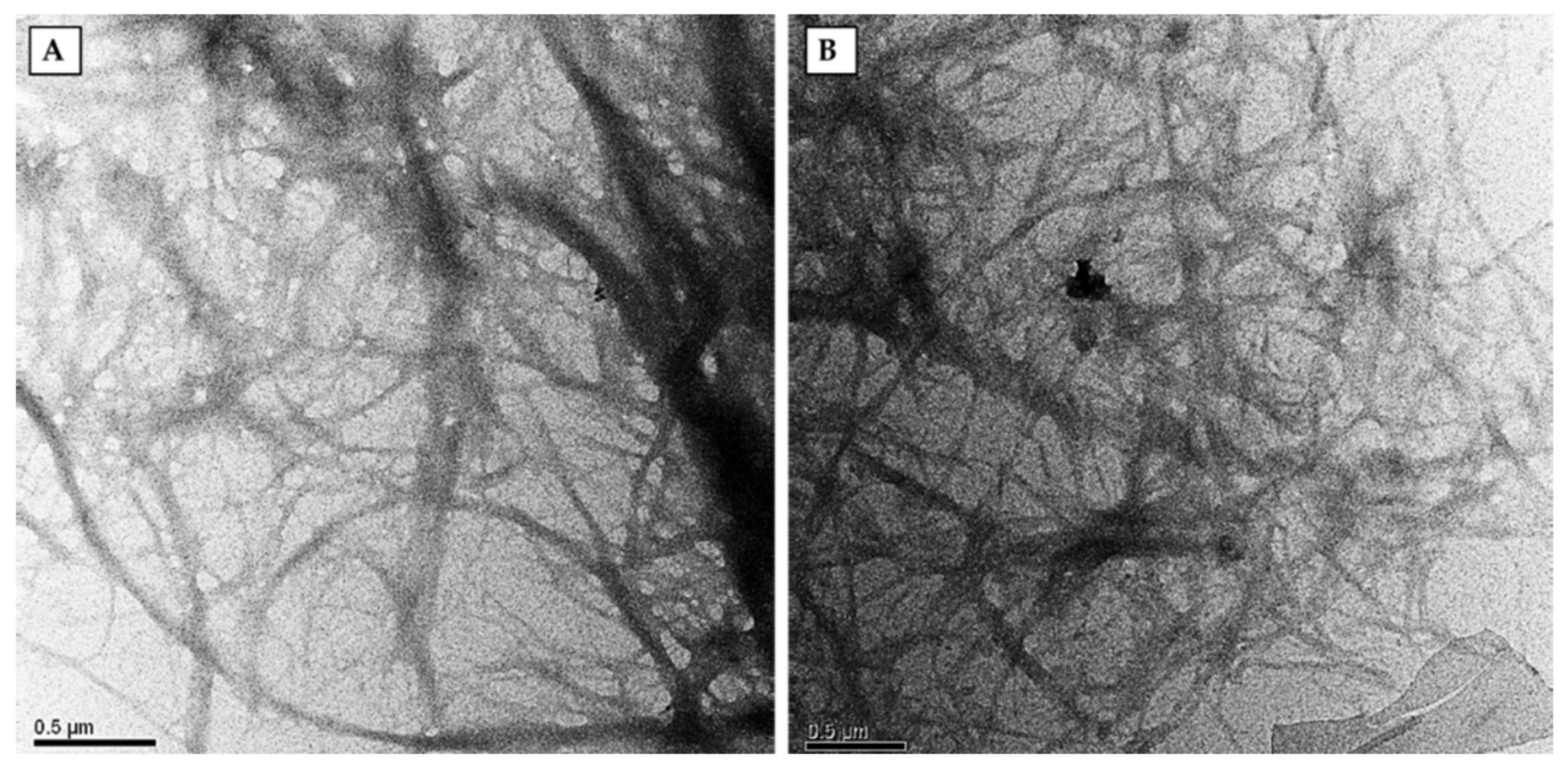

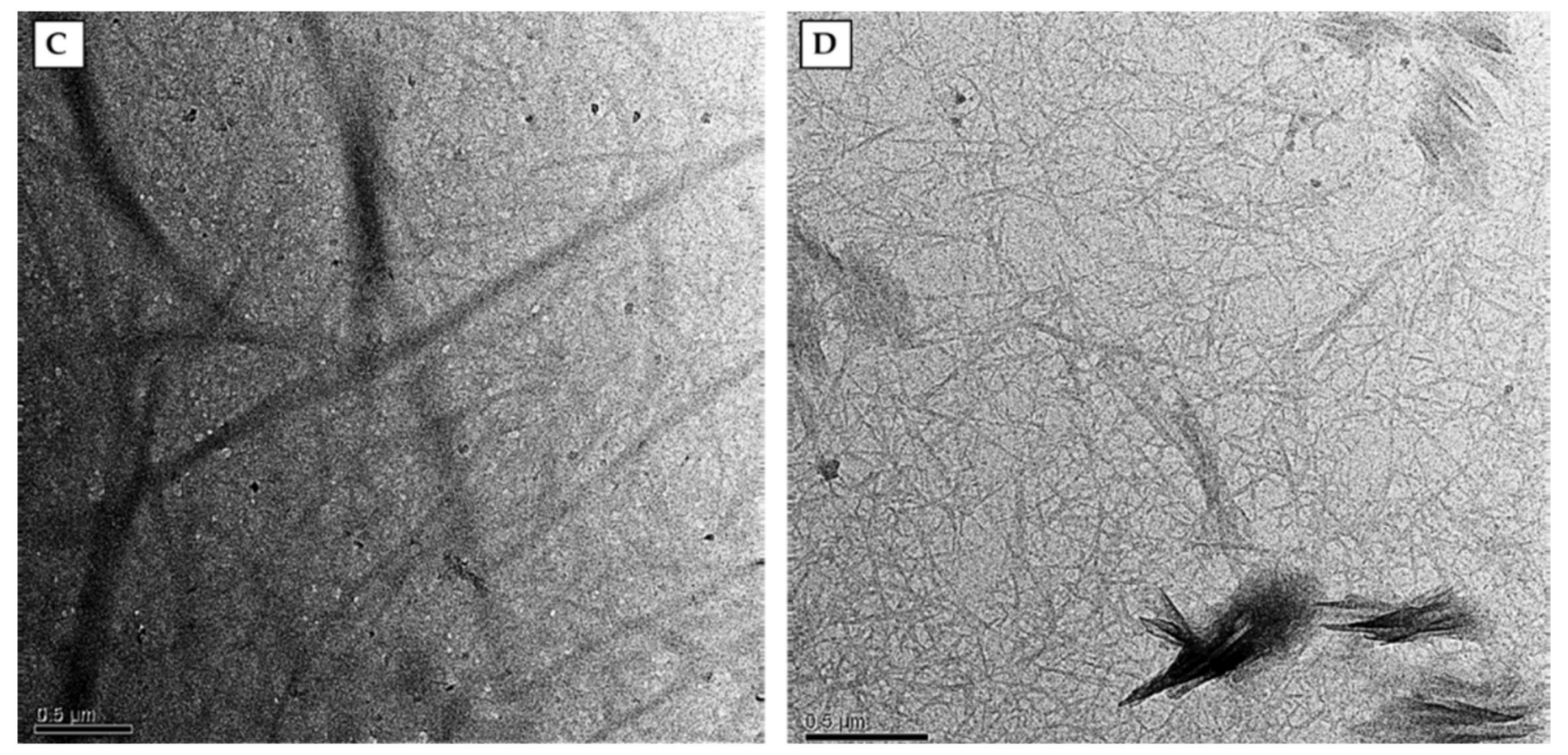
TEM images of virgin (**A**) and waste (**B**) enzymatic-treated cellulose nanofibers and of virgin (**C**) and waste (**D**) TEMPO-oxidised cellulose nanofibers.

Figure 2 shows the morphological characterisation by TEM analysis of the nanofibers obtained from both enzymatic (Figure 2A,B) and TEMPO-mediated oxidation (Figure 2C,D) processes. The images clearly show the formation in both cases of fibres with a width in the order of nanometres, as expected by the standard adopted procedures. Moreover, they confirm that analogous products can be obtained starting from both virgin (Figure 2A,C) and waste (Figure 2B,D) sources.

**Figure 3:**
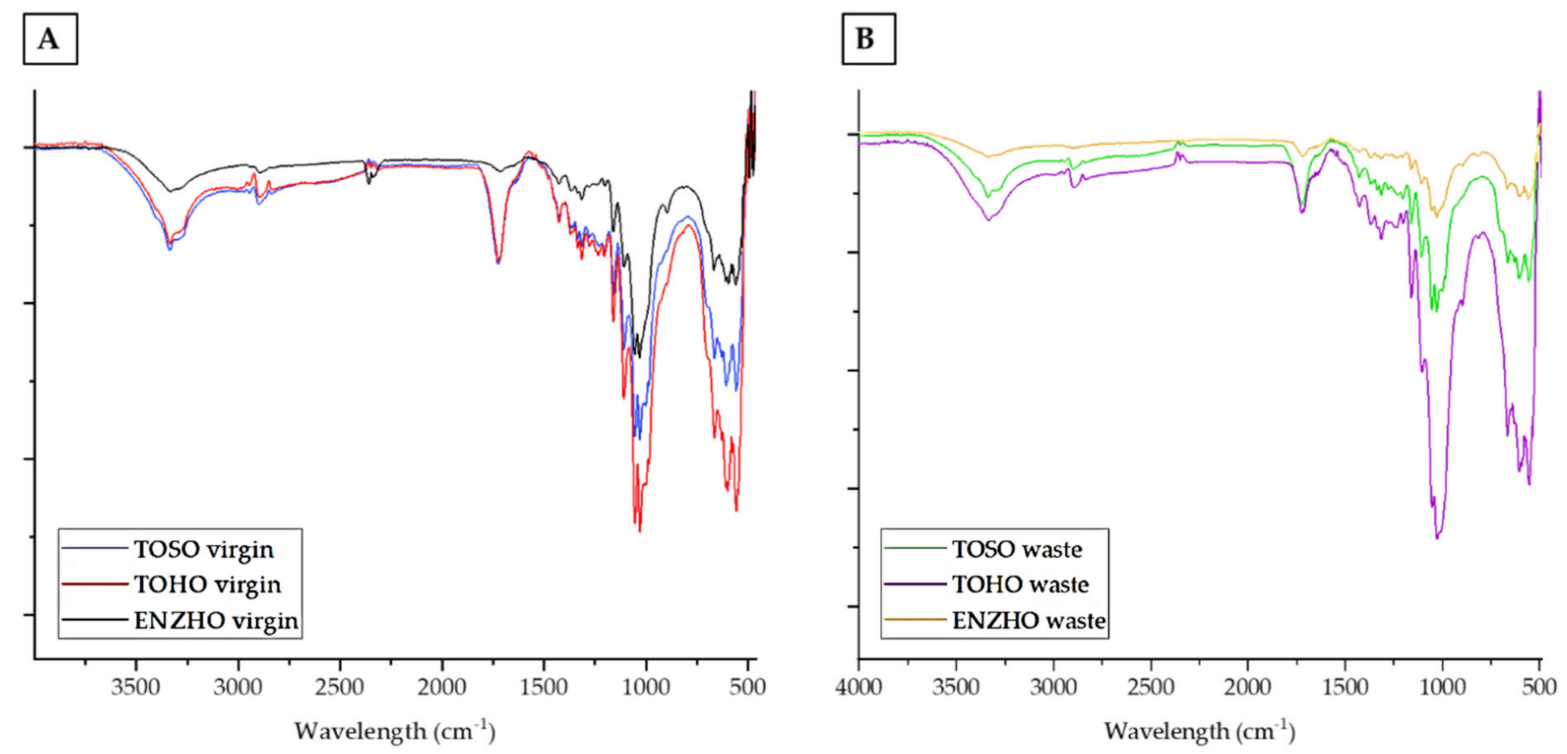
FTIR ATR spectra of virgin (**A**) and waste (**B**) cellulose nanofibers obtained with ENZHO, TOHO, and TOSO processes.

The FTIR ATR analysis of the six analysed samples highlights the main chemical differences among the nanofibers obtained following the ENZHO procedure and those derived from the TOHO and TOSO processes (Figure 3). It consists of the presence, for the oxidised ones, of the characteristic signal at 1725 cm^−1^, which refers to the C=O of the carboxylic moieties (Scheme 1A). Additionally, in this case, the identical trend is observed for nanofibres derived from both virgin (Figure 3A) and waste (Figure 3B) sources.

### 2.2. Life Cycle Impact Assessment and Interpretation

**Figure 4:**
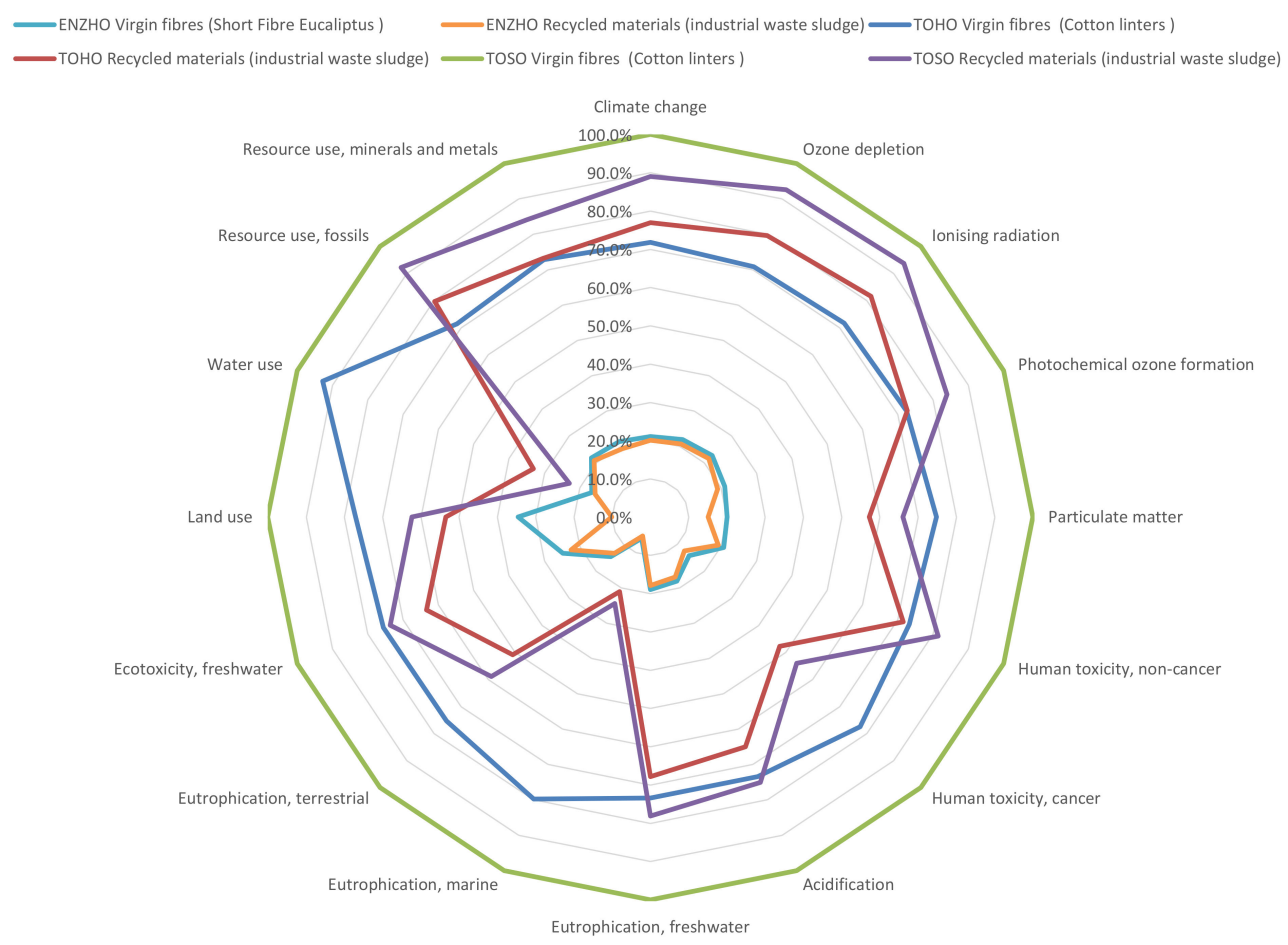
Relative environmental performances of the six CNFs based on the results of the characterisation step. The highest impact per each category is given 100% of the impact.

The impact assessment results of the six CNFs are fully reported in Table 1. An analysis of the whole set of results points out unequivocally that the TEMPO oxidation has a relevant effect on environmental performances. Indeed, for almost all impact categories, oxidised CNFs have evidently a far larger burden than non-oxidised ones. For the sake of clarity, a radar graph is shown in Figure 4 where the relative percentage impacts of the six CNFs are reported. As anticipated, non-oxidised CNFs have superior performances with respect to oxidised ones; this is due to the absence of chemicals and the reduced consumption of electricity (see Table 2 and Table 3); indeed, only enzymes are used in ENZHO processes, apart from water. 

For all production routes (see Figure 4), the use of recycled raw materials has, on average, a beneficial effect on the burden, counterbalanced by the lower initial content of cellulose (90% or more in the virgin raw material vs. 70% in the recycled one), which, in turn, entails a larger electricity consumption (Table 3). In the case of TOHO, indeed, there is a trade-off between these two factors, which is reflected in the worse performance of the processes from recycled materials with respect to the virgin source, at least in certain categories (see, for instance, climate change, ozone depletion, and resource uses of fossil fuels). In the case of TOSO, instead, the lower cellulose content does not affect sensibly the environmental burden, and the recycled material process is invariably more sustainable than the virgin one. 

Independently of the cellulose origin, the TOHO process is superior to TOSO in any category considered. The reason can be traced back to the lower energy consumption (Table 3); however, this fact deserves a bit of explanation. Sonication is a typical lab-scale activity, and as such, the energy consumption is not optimised; on the contrary, homogenisation was run on a somewhat larger instrument whose energy use was more efficient. 

In any case, the enzymatic route (ENZHO) seems to be the far-least impacting production process; of course, the product obtained is not oxidised and, as such, may have different uses and performances that, in cradle-to-the-grave LCA, shall be considered. 

In conclusion, from this first impact analysis, it seems that the preferred preparation method of oxidised CNFs is TOHO, and a further positive effect comes from the use of recycled raw materials. On the other hand, when the oxidation degree does not matter, the best choice is certainly the enzymatic route.

In the literature, there are few works addressing detailed LCA studies of nanocellulose materials from different preparation methods [28,31]. Unfortunately, the comparison of the results is often impaired by the impact assessment method chosen, as in the case of Li et al. [28]. However, despite the use of a completely different method, they too obtain that TOHO is better than TOSO at the laboratory scale.

A deeper look into the production route of oxidised CNFs highlights that all the chemicals and most of the energy are required by the pre-treatment, i.e., TEMPO oxidation, while step 2 consumes only water and a limited amount of electricity. This feature is clearly reflected in the impact’s distribution between the two steps (see Table 4); more than 90% of the impacts come from step 1 in each category. The opposite is true for the ENZHO route, where no chemicals are used, but the enzyme and only water and electrical energy are consumed. Indeed, it is step 2 that drives most of the environmental burden.

The EF3.0 impact assessment method also enables the normalisation and weighting (see Tables S2 and S3) steps in the impact assessment. Despite losing a bit of immediacy, it is possible to arrive at a single score and, consequently, have a direct comparison between the six options. It is worth noting that the set weighing factors is given more relevance only in climate change, i.e., about 20%; instead, all other categories contribute to the total impact by less than 10% each, without a specific predominance of one category over the others.

Looking at the total single scores (Table S3), the final outcome confirms the results obtained at the characterisation step: ENZHO as the best option, then TOHO and, last, TOSO. For each production method, the use of recycled cellulose is beneficial: the effect is tiny for ENZHO, more marked for TOHO, and definitely larger for TOSO.

### 2.3. Sensitivity Analysis

An in-depth analysis of the factors that have more influence on the environmental performances has revealed that energy consumption is on top. However, this is a well-known distorted effect of the lab scale, recognised by other authors [28,37,40,41]. 

In a recent work, Turk et al. [40] investigated the impacts of nanofibrillated cellulose from thermo-groundwood with different impact assessment methods, thus enabling at least a mild comparison of the results of the TOHO route presented here. Considering only the last two steps of their process, i.e., TEMPO oxidation and homogenisation, the climate change excluding biogenic carbon results in about 1.83–1.89% of the total impact (depending on the impact method), which would amount to around 0.148 kg CO_2_ eq per 10 g of CNF. In general, the impacts listed by Turk et al. were lower than those found in the present work for TOHO. This is explained by the different scales of analysis, as revealed by the energy consumption. It is acknowledged that CNF production is an energy-intensive activity in itself [31], and this feature is further aggravated when considering the lab-scale process [41]. Recognising this limit, Li et al. evaluated improvements in the impact assessments of CNF production by reducing the electrical energy consumption to about 8.3% of the laboratory estimated amount [28]. An engineering-based approach was adopted by Bartolozzi et al. [37] to create the up-scale scenario.

Recognising that the electrical energy consumption is largely overestimated at the lab scale, and in view of its dramatic influence on the final impacts, a sensitivity analysis was performed. The total energy consumption was reduced by 50% (scenario 2) and 75% (scenario 3) with respect to the base scenario (1). Detailed characterisation results are in the Supporting Materials (Tables S4 and S5). Normalisation and single-score results were not reported for brevity but, by using the factors of Table S1, can be easily calculated.

**Figure 5:**
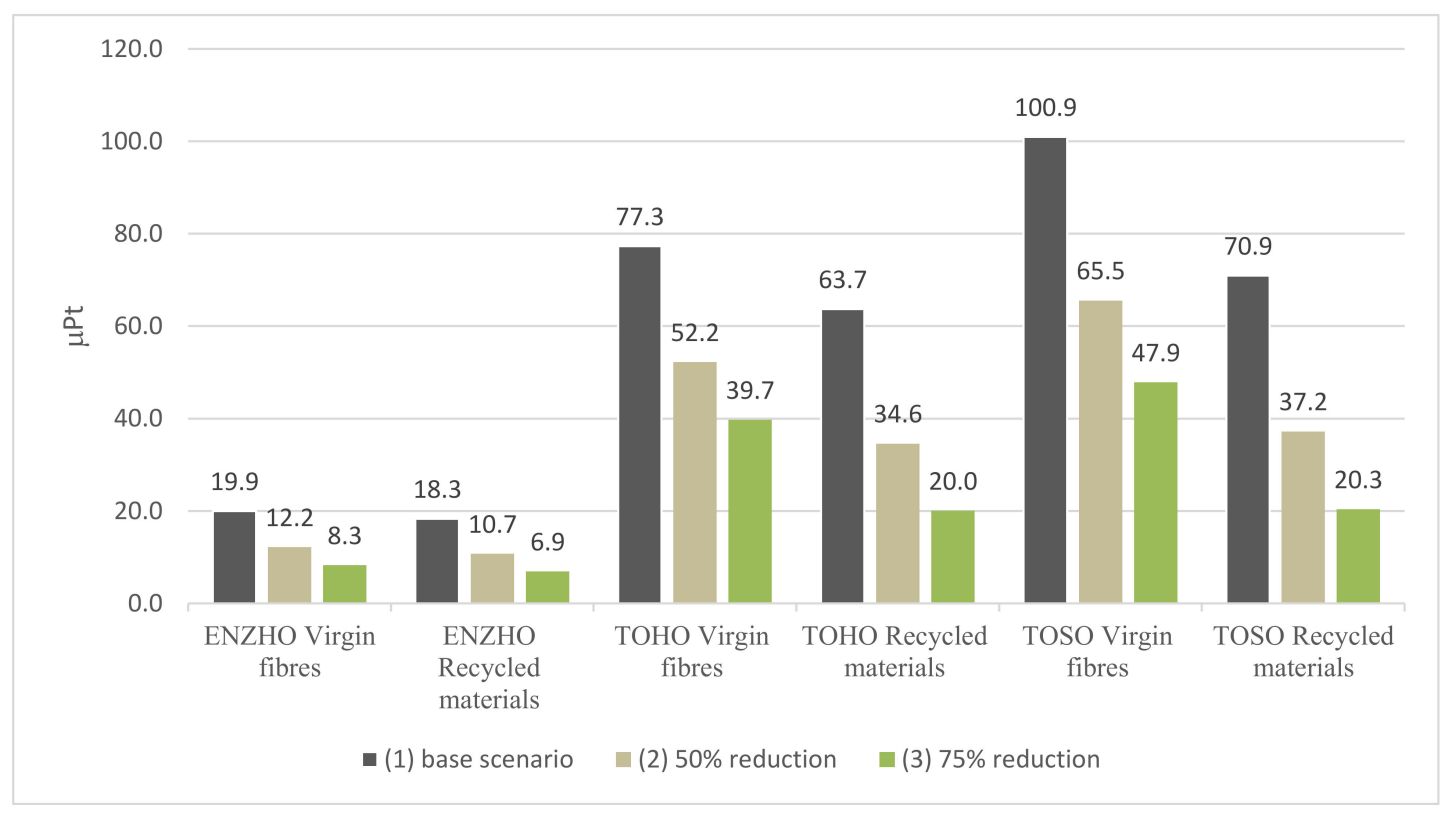
Single scores of the six CNFs in three scenarios: base scenario (1), the reduction of electrical energy consumption by 50% scenario (2), and the reduction of electrical energy by 75% scenario (3).

The electrical energy reduction does not affect the qualitative results: ENZHO is always the best performing route, followed by TOHO and, last, by TOSO. In the same way, recycled cellulose has a beneficial effect in all three methods. Instead, the impacts change substantially from a quantitative point of view. The trend is clearly shown in Figure 5, where single scores decrease drastically as a consequence of the energy reduction. Moreover, differences between the TOHO and TOSO routes are progressively cancelled upon reducing the electrical energy.

## 3. Materials and Methods

### 3.1. Cellulose Sources

The materials used for experimentation included both virgin and waste sources. Fibre lengths of all cellulose sources were obtained by fibre image analysis with a MorFi Techpap (Saint Martin d’Hères, France) Fibre and Shive analyser. The virgin category included, at first, cotton linters from the Bartoli paper mill (Capannori, Lucca, Italy). This fibre consists of 95% pure cellulose, with 5% impurities. The length of the fibre was between 2 and 6 mm, while the diameter was between 17–27 μm. The degree of refinement of the fibre (Schopper-Riegler Grade, °SR) was 12. The other virgin fibre used was a bleached mixed hardwood kraft pulp obtained from Burgo Group (Altavilla Vicentina, Vicenza, Italy). This fibre had a low lignin content (cellulose content about 90%) and showed a fibre length between 500 μm and 1 mm, while the fibre diameter was again around 20 μm. The °SR of this fibre was 16. 

Among the recycled fibres, we selected an industrial waste sludge containing 70% cellulose fibres. These fibres were of lower quality than those found in virgin sources, and they had a significantly shorter length compared to the cotton linters (around 700 μm). The recycled materials showed a °SR significantly higher (26) in comparison with the °SR of virgin fibres. Table 5 summarises the main characteristics of the selected sources.

### 3.2. Cellulose Nanofibres Production

All the reagents were purchased from Sigma-Aldrich (Milano, Italy). Deionised water was produced with a Millipore Elix^®^ deioniser with Progard^®^ S2 ion exchange resins (Merck KGaA, Darmstadt, Germany). Endoglucanase FiberCare R was provided by Novozymes (Bagsværd, Denmark). Other equipment used in the procedures included a Branson Sonifier 250 equipped with a 6.5-mm probe tip (Fisher Scientific Italia, Rodano (MI), Italy), a GEA Niro Soavi NS3006L and high-pressure homogeniser (Parma, Italy), and a PFI refiner (Testing Machines Inc., New Castle, DE, USA), compliant with the UNI EN ISO 5264-2:2011 normative. FTIR ATR characterisation of the cellulose nanofibers was performed with a 640-IR FTIR spectrometer from Agilent Technologies (Santa Clara, CA, USA). The morphological characterisation of TOUS-CNFs was obtained by a transmission electron microscope (TEM; Philips CM 200, Koninklijke Philips N.V., Amsterdam, The Netherlands) operating at 200 kV and equipped with a field emission gun filament.

**Scheme 2:**
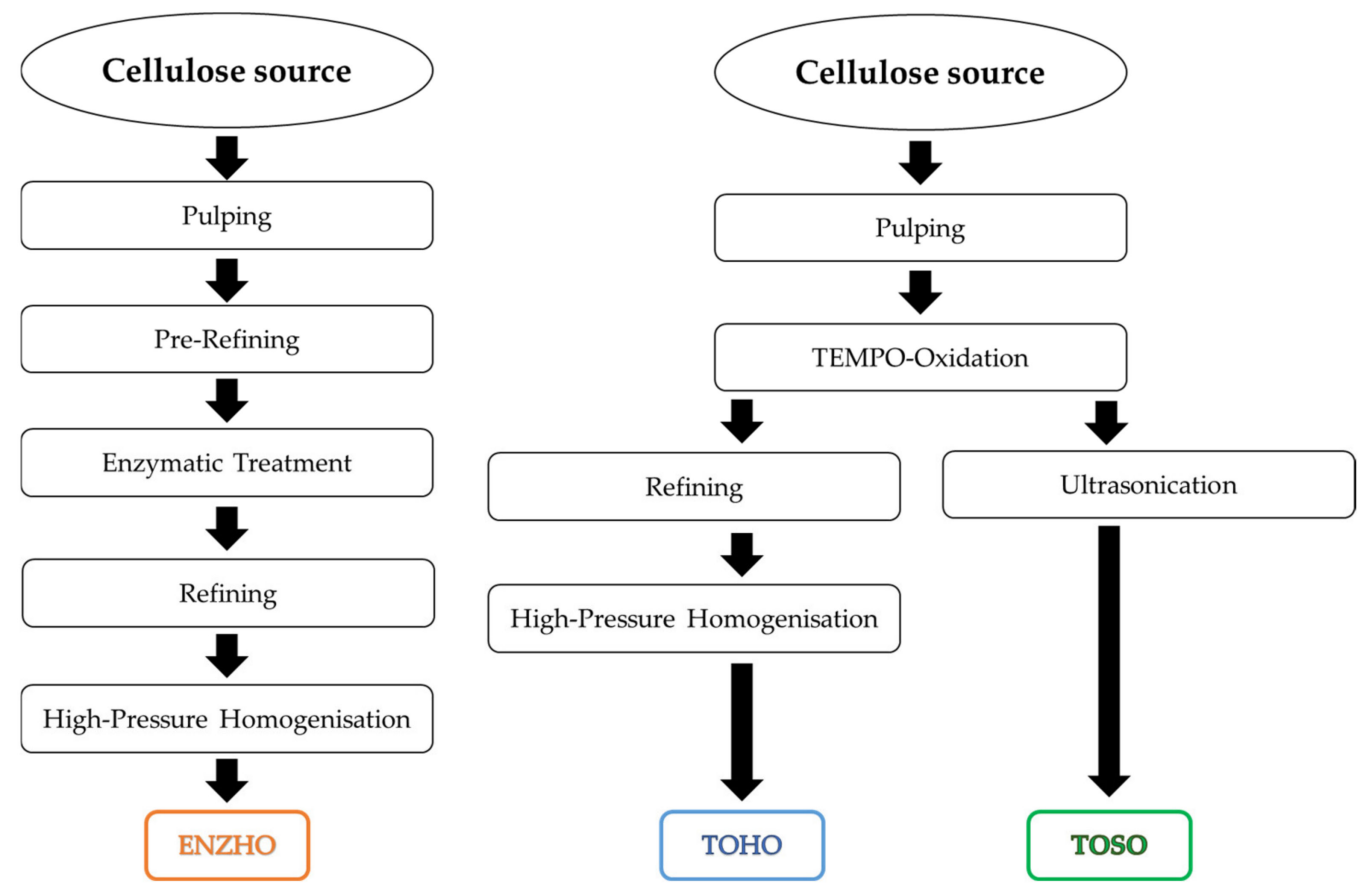
Processes for the preparation of the three different types of cellulose nanofibres.

Cellulose nanofibres (CNF) were produced following 3 different processes: ENZHO (Enzymatic Treatment + Homogenisation), TOHO (TEMPO-Oxidation + Homogenisation), and TOSO (TEMPO-Oxidation + Ultrasonication). All these processes will be described in detail in the following sections. Scheme 2 summarises all the processes to obtain the different types of cellulose nanofibres. The starting quantity used was always 10 g (lab scale), except for the use of the high-pressure homogeniser, which, due to the nature of the instrument, required working on quantities equal to 100 g per batch (semi-pilot scale).

#### 3.2.1. ENZHO Nanofibres

The first step to produce ENZHO non-oxidised cellulose nanofibres is the evaluation of the initial degree of refining in accordance with Method UNI EN ISO 5267-1:2002 (Schopper-Riegler method). A suspension at a fibre concentration of 1.5% *w*/*w* in deionised water is first prepared, and a volume corresponding to 2 g of dry cellulose is then taken, diluted to exactly 1 L with deionised water and subjected to the evaluation of the Schopper-Riegler degree. The suspension is drained naturally on a special metal cloth, where the water excess falls laterally and is collected and measured in a cylinder graduated in the SR scale. If the SR degree is lower than 20, a pre-refining process is needed; otherwise, the enzymatic treatment is carried out directly. 

If needed, the purpose of the pre-refining treatment is to obtain a SR degree of about 25–30 °SR. The starting cellulosic material is used to obtain an aqueous suspension at a concentration of about 10% with the aid of a pulper. The mixture is subjected to mechanical refining using a PFI-type refiner setting of 1000 rotor revolutions. Pre-refined cellulose pulp then undergoes an enzymatic treatment using the endoglucanase FiberCare R. The enzymatic reaction is carried out in a reactor at a controlled temperature of 50 °C for 1 hour at a 2% *w*/*w* concentration, keeping the suspension under agitation by a mechanic stirrer. The concentration of the endoglucanase is 0.1 mg/g. After the reaction, the suspension is immediately filtered to block enzyme activity. 

The material is then resuspended in deionised water at a 10% *w*/*w* concentration for the refining phase [42], through which the cellulosic suspension is refined up to a SR degree of 75–80 (maximum limit of cellulose refining that can be obtained with laboratory instruments). Additionally, in this case, we used the PFI instrument, reaching a °SR of 75–80 with 5000 rotor revolutions of the refiner.

After the refining treatment, the cellulose pulp is homogenised [18]. Each sample is prepared in an aqueous suspension of 6 L at a fibre concentration of 2% *w*/*w*, maintained at room temperature. 

The cycles are conducted at different operating pressures:

- 1 cycle at 500 bar
- 1 cycle at 1000 bar
- 2 cycles at 1250/1300 bar
- 3 cycles at 1400/1500 bar (maximum operating pressure)

for a total of 7 discontinuous cycles, followed by a final recovery of the 2% *w*/*w* non-oxidised nanocellulose suspension.

#### 3.2.2. TOHO Nanofibres

For the synthesis of TOHO-oxidised cellulose nanofibres, the first step is the TEMPO-mediated oxidation [16,17]. The selected cellulose source is suspended in deionised water at a concentration of 5% *w*/*w* by means of a pulper. Simultaneously, in a keg placed under a mechanic stirrer, tetramethyl-piperidine-N-oxide (TEMPO) and KBr are dissolved in a small amount of deionised water. Once the paper results are homogeneously pulped in water, the suspension is transferred into the keg, and water is added in order to obtain a final concentration of 2% *w*/*w*. While keeping the solution stirred, a pH meter and two dropping funnels are installed above the keg, one of the funnels containing an aqueous solution of NaClO and the other one with NaOH 4 M. NaClO is then slowly dripped into the cellulose suspension, and the pH is monitored to keep it above 10.5–11 by dripping NaOH 4 M; then, the solution is left stirring overnight. After 12–16 hours, the oxidised cellulose is acidified with concentrated HCl for aggregation of the cellulose fibres and their easy separation from water. The oxidised cellulose is then filtered onto a Büchner funnel equipped with a tissue filter and washed with deionised water until neutrality. The refining phase and the homogenisation treatment are conducted by the same procedure as described above, obtaining a TEMPO-oxidised 2% *w*/*w* nanocellulose suspension.

#### 3.2.3. TOSO Nanofibres

For the synthesis of TOSO-oxidised cellulose nanofibres, the first step is TEMPO-mediated oxidation, carried out as described in the previous paragraph.

Oxidised cellulose pulp from the TEMPO-oxidation treatment is then suspended in deionised water in order to obtain a 2% *w*/*w* concentration for the ultrasonication treatment. To this suspension, granular NaOH is added, getting a basic pH (the amount of NaOH is calculated according to the oxidation rate of the cellulose pulp). The suspension is sonicated with an immersion ultra-sonifier, cooling it with an ice-bath to promote the separation of these fibres. After a few minutes of ultrasonication, we obtain a 2% *w*/*w* suspension of TEMPO-oxidised cellulose nanofibers.

#### 3.2.4. Titration of TOSO and TOHO Cellulose Nanofibers

To estimate the concentration of carboxyl groups on the cellulose structure after oxidation, titration was performed with NaOH using phenolphthalein as the colorimetric indicator. To begin with, an NaOH solution was titrated by means of potassium hydrogen phthalate. A solution was prepared by dissolving phthalate in water and by adding 2 to 3 drops of phenolphthalein solution 10 mM in acetonitrile. Then, the NaOH solution was dripped into the beaker under continuous stirring until neutralisation of the phthalate to calculate the NaOH concentration. The previously titrated NaOH solution was then used to titrate the oxidised cellulose obtained from the TOHO and TOSO processes. A TOCNF water dispersion was prepared by adding TOCNF to deionised water and sonicating it to improve the grade of dispersion. A drop of phenolphthalein solution was added. The NaOH solution previously titrated was then dripped into the beaker under continuous stirring until neutralisation of the carboxyl acids of the oxidised cellulose. The concentration of the carboxyl groups was calculated by means of the following equation:

$$\left[COOH\right]=\frac{M_{NaOH,sol}\times x_{COOH}\times V_{NaOH,sol}}{m_{TOCNF}}$$

where $M_{NaOH,sol}$ is the moles of NaOH in 1 L of the solution, $x_{COOH}$ is the molar fraction of COOH per mol of NaOH, $V_{NaOH,sol}$ is the dripped volume of NaOH solution, and $m_{TOCNF}$ is the titrated mass of TOCNF.

### 3.3. Life Cycle Assessment (LCA) Methodology

The LCA developed here is compliant with the ISO standards [21,22], whose structure is made of four stages: goal and scope, life cycle inventory (LCI), life cycle impact assessment (LCIA), and life cycle interpretation. The first two stages, i.e., goal and scope and LCI, are discussed in the next sub-sections, being more methodological and related to data collection. Instead, the last two stages, LCIA and Life Cycle Interpretation, are discussed in Section 2, i.e., Results and Discussion. 

#### 3.3.1. Goal

Different preparation routes, as well as different raw materials, are possible for nanocellulose production, both pristine and oxidised; the goal of the present work is to estimate from an environmental point of view which options are more sustainable. For this reason, an LCA study is performed with the intent of comparing the environmental performances of the different preparation routes developed at the laboratory scale. The analysis also has the ambition of supplying reliable information to people that will have to make an informed decision at the industrial scale. For this reason, there will also be a discussion about the scale-up results. Consistent with this goal, the LCA is of an attributional type [43,44], and the scenario is from-cradle-to-the-gate.

#### 3.3.2. Functional Unit

The functional unit (FU) adopted is 10 g of cellulose nanofibres (CNF) in 2%wt water suspension produced in the laboratory to be used in future applications. The quantified amount of FU is consistent with the scale of the experiments and analogous to that assumed by Li et al. [28]. It is important to highlight that the final use of the CNF is not yet explicit at this level of the study and cannot be. Nanocellulose can play a relevant role in the development of a large number of sustainable products, as pointed out in a recent review [25]. For instance, some authors tested CNF-derived materials as additives for the building sector [45] or sorbents for water treatment [46]. 

#### 3.3.3. System Boundary

The product system investigated here is the production at the lab scale of 10 g of CNF starting from virgin raw materials (hardwood kraft pulp and cotton linters) and secondary raw materials (industrial waste sludge-derived cellulose). Details of the CNF production routes were given in Section 2.1.

**Figure 6:**
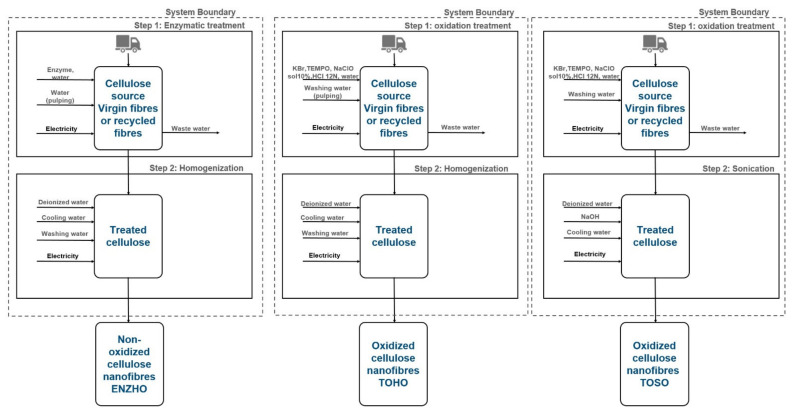
System boundaries of the three production processes: ENZHO, TOHO, and TOSO.

The scenario of the analysis is from-cradle-to-the-gate, where the gate is the final product obtained in the laboratory. The system boundary is schematically reported in Figure 6, encompassing:

all the operations carried out in the laboratory, i.e., CNF production from cellulose sources, including energy and water uses;

- synthesis of all chemicals used in the above-mentioned processes, i.e., ancillary products;
- virgin raw material production;
- transport of raw materials from suppliers to the lab, considering a reference distance of 200 km for all materials;
- transports of chemicals from supplier to consumer by using “market for” datasets from the database Ecoinvent 3.6 (https://www.ecoinvent.org/support/faqs/methodology-of-ecoinvent-3/what-is-a-market-and-how-is-it-created.html, last accessed 20 March 2021).

Instead, waste treatment processes of input recycled products, assimilated with burden-free raw materials, are neglected; in other words, the recycling impact is fully allocated to the system product producing the recycled materials, often referred to as the 0–100 approach [47].

These last assumptions are consistent with the goal of the study, which is primarily focused on estimating the best preparation route. The influence of raw material transport distances can be more relevant at the industrial scale, but in that case, a mandatory prerequisite would be the knowledge of the plant location, and this is certainly beyond the aim of this work. A similar approach has been adopted in other recent LCA studies of nanocellulose products [39,40].

#### 3.3.4. Data Requirements

Chemicals amounts, as well as water and energy consumptions, are primary data. These data are the results of experimental activity carried out in the laboratories of Politecnico di Milano and of Innovhub—Stazioni Sperimentali per l’Industria S.r.l. (https://www.innovhub-ssi.it/, last accessed 19 March 2021).

Chemicals, raw materials, and electrical energy production processes are secondary data, whose source is the Ecoinvent 3.6 database (https://www.ecoinvent.org/, last accessed 19 March 2021), as implemented in the software Simapro 9.1.1 (https://simapro.com/2020/whats-new-in-simapro-9-1-1/, last accessed 19 March 2021). A detailed list of the unit processes used is reported in Table 6. No allocation procedure is necessary.

#### 3.3.5. Life Cycle Impact Assessment (LCIA) Methods

The impact assessment is estimated by the EF 3.0 method developed for the Environmental Footprint initiative [48]. For this impact assessment method, both the normalisation and weighing factors are available for 16 impact categories (for convenience, their values are reported in the Supplementary Materials Table S1), although there are characterisation factors for the 28 impact categories. However, in the present analysis, only the first 16 categories with normalisation and weighing factors are considered. This is the reason why the EF 3.0 impact assessment method was preferred over others, because it enables to also perform the optional elements of the LCIA [22], while other methods do not. This possibility is considered highly relevant in this study because of the specific goal; indeed, single scores offer an additional tool in the decision process. 

### 3.4. Life Cycle Inventory (LCI)

Primary data come from the laboratory activity. In total, 6 different CNFs were produced via three different routes: ENZHO, TOHO, and TOSO. For each route, a virgin and a recycled raw material were used.

The complete inventory of the materials, chemicals, and water used is reported in Table 2. For the sake of completeness, the cellulose contents in the starting raw materials and oxidation degree in TEMPO-oxidised CNF are given. The electrical energy consumptions are reported in Table 3.

## 4. Conclusions

The LCA at the lab scale clearly shows how the ENZHO approach is significantly more convenient in terms of sustainability, compared with the TOHO and TOSO processes, remarking how the TEMPO-oxidation step highly impacts the overall process. 

Nevertheless, the different chemical structures of the CNF obtained following the two different approaches (the enzymatic and the oxidation ones) should be stressed. The presence of carboxylic units on the CNF backbone imparts additional properties, which open the way to new or more convenient solutions. As examples:

i. Nanocellulose-based composites: Carboxylic moieties are suitable groups that can be valorised for further functionalisation of the CNF or for a simple crosslinking of nanofibres in nanostructured systems, avoiding the use of additional chemicals, which are instead required in the case of CNF obtained following the ENZHO approach [49].
ii. Nanocellulose-based additives: CNF-bearing carboxylic units show different properties when used as additives, for example, in construction materials, varying with the bases of the oxidation degree. These aspects can include a better interaction with the matrix and a modification of rheological properties, water retention, and so on [44].
iii. Nanocellulose-based hydrogels: It has been reported how the interaction between the polyvalent cations and the carboxylic units leads to the formation of stable hydrogels with tunable rheological properties, which find biomedical applications, such as drug delivery systems [50].

The sensitivity analysis emphasised the high impact of electricity consumption at the lab scale, which can be significantly reduced when modelling a pilot-scale process. While this approach confirmed ENZHO as the best-performing approach, the differences between TOHO and TOSO were almost delated. However, it should also be considered that a scale-up of ultrasonication treatment on large volumes is far from easily realised, in contrast to the homogenisation process, which is already used on the industrial scale for different productions.

Finally, while using recycled sources as raw materials appears to be the ideal choice for supporting the sustainability of the processes, particular attention should be paid to the real content of the cellulose present in the selected matrices, as a low percentage of cellulose content could neutralise the benefits of the original virtuous choice.

## Figures and Tables

**Table 1:** Life cycle impact assessment of the six CNFs produced at the lab scale; the results are given per FU and refer to the characterisation stage.

Impact Category	Unit	Enzymatic Treatments & Homogenisation (ENZHO)		TEMPO-Oxidation and Homogenisation (TOHO)		TEMPO-Oxidation and Ultrasonication (TOSO)	
		Virgin Materials (Hardwood Kraft Pulp)	Recycled Materials (Industrial Waste Sludge)	Virgin Materials (Cotton linters)	Recycled Materials (Industrial Waste Sludge)	Virgin Materials (Cotton Linters)	Recycled Materials (Industrial Waste Sludge)
Climate change	kg CO_2_ eq	1.86 × 10^−1^	1.77 × 10^−1^	6.32 × 10^−1^	6.78 × 10^−1^	8.80 × 10^−1^	7.84 × 10^−1^
Ozone depletion	kg CFC11 eq	2.54 × 10^−8^	2.40 × 10^−8^	8.21 × 10^−8^	9.25 × 10^−8^	1.16 × 10^−7^	1.08 × 10^−7^
Ionising radiation	kBq U-235 eq	2.31 × 10^−2^	2.19 × 10^−2^	7.28 × 10^−2^	8.28 × 10^−2^	1.02 × 10^−1^	9.52 × 10^−2^
Photochemical ozone formation	kg NMVOC eq	4.52 × 10^−4^	4.07 × 10^−4^	1.56 × 10^−3^	1.56 × 10^−3^	2.14 × 10^−3^	1.80 × 10^−3^
Particulate matter	disease inc.	5.13 × 10^−9^	3.86 × 10^−9^	1.91 × 10^−8^	1.46 × 10^−8^	2.56 × 10^−8^	1.69 × 10^−8^
Human to xicity, noncancer	CTUh	1.93 × 10^−9^	1.79 × 10^−9^	6.82 × 10^−9^	6.67 × 10^−9^	9.31 × 10^−9^	7.58 × 10^−9^
Human toxicity, cancer	CTUh	7.32 × 10^−11^	6.40 × 10^−11^	3.98 × 10^−10^	2.45 × 10^−10^	5.13 × 10^−10^	2.78 × 10^−10^
Acidification	mol H+ eq	1.02 × 10^−3^	9.55 × 10^−4^	4.13 × 10^−3^	3.65 × 10^−3^	5.62 × 10^−3^	4.22 × 10^−3^
Eutrophication, freshwater	kg P eq	5.42 × 10^−5^	5.12 × 10^−5^	2.10 × 10^−4^	1.94 × 10^−4^	2.87 × 10^−4^	2.24 × 10^−4^
Eutrophication, marine	kg N eq	1.59 × 10^−4^	1.42 × 10^−4^	2.11 × 10^−3^	5.59 × 10^−4^	2.65 × 10^−3^	6.47 × 10^−4^
Eutrophication, terrestrial	mol N eq	1.94 × 10^−3^	1.77 × 10^−3^	1.00 × 10^−2^	6.76 × 10^−3^	1.33 × 10^−2^	7.82 × 10^−3^
Ecoto× icity, freshwater	CTUe	4.40	3.99	13.0	11.0	18.0	13.1
Land use	Pt	3.52	1.03	7.84	5.44	10.0	6.34
Water use	m^3^ depriv.	3.89 × 10^−1^	3.65 × 10^−1^	2.17	7.73 × 10^−1^	2.33	5.36 × 10^−1^
Resource use, fossils	MJ	2.70	2.58	8.84	9.86	12.0	11.4
Resource use, minerals, and metals	kg Sb eq	1.57 × 10^−6^	1.42 × 10^−6^	5.36 × 10^−6^	5.39 × 10^−6^	7.37 × 10^−6^	6.20 × 10^−6^

**Table 2:** Inventory of the chemicals, water, and raw materials for the six CNFs produced at the lab scale; amounts are given per FU.

Materials and Chemicals	Unit	Enzymatic Treatments and Homogenisation (ENZHO)		TEMPO-Oxidation and Homogenisation (TOHO)		TEMPO-Oxidation and Ultrasonication (TOSO)	
		Virgin Materials (Hardwood Kraft Pulp)	Recycled Materials (Industrial Waste Sludge)	Virgin Materials (Cotton Linters)	Recycled Materials (Industrial Waste Sludge)	Virgin Materials (Cotton Linters)	Recycled Materials (Industrial Waste Sludge)
***Step 1—input***							
Raw material	g	11.08	11.08	10.61	12.51	12.88	14.70
Cellulose Content	%	90	70	95	70	95	70
Deionised Water	L	0.54	0.44	0.6	0.71	0.73	0.83
FibreCare R (Novozymes)	mg	1.3	1.3	-	-	-	-
Potassium Bromide	g	-	-	1.64	1.93	1.98	2.27
TEMPO	g	-	-	0.23	0.27	0.27	0.32
NaClO (solution 10%)	mL	-	-	35.02	68.79	43.04	80.88
NaOH solution 4N	mL	-	-	0.44	1.56	3.87	1.84
HCl solution 12N	mL	-	-	0.35	1.04	2.57	1.23
Washing water	L	-	-	0.53	0.63	0.64	0.74
***Step 1—output***							
Treated Cellulose	g	10.52	10.52	10.53	10.63	12.5	12.5
Wastewater	L	0.54	0.44	1.13	1.33	1.38	1.57
***Step 2—input***							
Treated Cellulose		10.52	10.52	10.53	10.52	12.5	12.5
NaOH seeds	g	-	-			0.515	0.53
Deionised water	L	0.52	0.52	0.42	0.42	0.5	0.50
Cooling water	L	5.54	5.54	4.42	5.21	1.25	1.25
Washing water	L	-	-	3.54	3.13	-	-
***Step 2—output***							
**Nanocellulose**	g	10	10	-	-	-	-
**Oxidised Nanocellulose**	g	-	-	10	10	10	10
**Oxidation rate**	mmol_COOH_/g_TOCNF_	-	-	1.0450	1.0034	1.0310	1.0034

**Table 3:** Electrical energy consumption for the six CNFs produced at the lab scale; amounts are given per FU.

Materials and Chemicals	Unit	Enzymatic Treatments and Homogenisation (ENZHO)		TEMPO-Oxidation and Homogenisation (TOHO)		TEMPO-Oxidation and Ultrasonication (TOSO)	
		Virgin Materials (Hardwood Kraft Pulp)	Recycled Materials (Industrial Waste Sludge)	Virgin Materials (Cotton Linters)	Recycled Materials (Industrial Waste Sludge)	Virgin Materials (Cotton Linters)	Recycled Materials (Industrial Waste Sludge)
***Step 1—input***							
Electricity (pulping)	Wh	4.6	4.6	3.67	4.33	5.35	5.09
Electricity (pre-refining)	Wh	10.12	-	-	-	-	-
Electricity (stirring)	Wh	83.1	83.1	1194.16	1406.98	1739.69	1654.41
Electricity (pumping)	Wh	6.85	6.85	65.46	77.12	95.36	90.69
Electricity—step 1	Wh	104.67	94.55	1263.29	1488.43	1840.4	1750.19
	%	25.0%	23.1%	92.4%	94.9%	96.4%	96.2%
***Step 2—input***							
Electricity (refining)	Wh	10.12	10.12	8.08	-	-	-
Electricity (homogenisation)	Wh	304.71	304.71	95.154	80.76	-	-
Electricity (sonication)	Wh	-	-	-	-	69	69
Electricity—step 2	Wh	314.83	314.83	103.234	80.76	69	69
		75.0%	76.9%	7.6%	5.1%	3.6%	3.8%
Electricity–total	Wh	419.5	409.38	1366.524	1569.19	1909.4	1819.19

**Table 4:** Life cycle impact assessment of the six CNFs produced at the lab scale: percentage contribution of step 1 with respect to the whole process.

Impact Category	Unit	Enzymatyc Treatments and Homogenisation (ENZHO)		TEMPO-Oxidation and Homogenisation (TOHO)		TEMPO-Oxidation and Ultrasonication (TOSO)	
		Virgin Fibres (Hardwood Kraft Pulp)	Recycled Materials (Industrial Waste Sludge)	Virgin Fibres (Cotton Linters )	Recycled Materials (Industrial Waste Sludge)	Virgin Fibres (Cotton Linters )	Recycled Materials (Industrial Waste Sludge)
Climate change	kg CO_2_ eq	27%	23%	94%	94%	97%	96%
Ozone depletion	kg CFC11 eq	28%	23%	93%	95%	96%	96%
Ionising radiation	kBq U-235 eq	27%	23%	92%	94%	96%	96%
Photochemical ozone formation	kg NMVOC eq	31%	23%	94%	94%	97%	96%
Particulate matter	disease inc.	43%	24%	95%	94%	97%	96%
Human toxicity, non-cancer	CTUh	30%	24%	92%	93%	97%	96%
Human toxicity, cancer	CTUh	33%	23%	95%	92%	98%	96%
Acidification	mol H+ eq	28%	23%	95%	94%	97%	96%
Eutrophication, freshwater	kg P eq	28%	23%	94%	94%	97%	96%
Eutrophication, marine	kg N eq	35%	28%	99%	95%	99%	96%
Eutrophication, terrestrial	mol N eq	30%	23%	96%	94%	98%	96%
Ecotoxicity, freshwater	CTUe	41%	35%	91%	90%	93%	91%
Land use	Pt	78%	23%	97%	96%	98%	97%
Water use	m^3^ depriv.	13%	7%	82%	49%	96%	82%
Resource use, fossils	MJ	27%	23%	93%	94%	96%	96%
Resource use, minerals, and metals	kg Sb eq	31%	23%	93%	94%	96%	96%

**Table 5:** Fibre length and °SR of selected cellulose sources for the production of the nanofibers.

Source	Category	Fiber Length (mm)	°SR
Cotton linters	Virgin	2–6	12
Hardwood kraft pulp	Virgin	0.5–1	16
Industrial waste sludge	Waste	0.5–1	26

**Table 6:** Modelling and sources of the secondary data.

Inputs	Description	Source
Raw material: hardwood kraft pulp	Proxy datum: Sulphate pulp, bleached {RER}|market for sulphate pulp, bleached|Cut-off, U	Ecoinvent 3.6
Raw material: cotton linters	Proxy datum: Fibre, cotton {GLO}|market for fibre, cotton|Cut-off, U	Ecoinvent 3.6
Raw material: Industrial waste sludge	Waste: burden free	-
Deionised water	Water, deionised {Europe without Switzerland}|market for water, deionised|Cut-off, U	Ecoinvent 3.6
FibreCare R (NovoEnzymes)	Proxy datum: Enzymes {GLO}|market for enzymes|Cut-off, U	Ecoinvent 3.6
Potassium Bromide	Proxy datum: Potassium chloride, industrial grade {GLO}|market for potassium chloride, industrial grade|Cut-off, U	Ecoinvent 3.6
TEMPO	Proxy datum:Piperidine {GLO}|market for|Cut-off, U	Ecoinvent 3.6
NaClO (solution 10%)	Sodium hypochlorite, without water, in 15% solution state {RER}|market for sodium hypochlorite, without water, in 15% solution state|Cut-off, U	Ecoinvent 3.6
NaOH solution 4N	Sodium hydroxide, without water, in 50% solution state {GLO}|market for|Cut-off, U	Ecoinvent 3.6
HCl solution 12N	Hydrochloric acid, without water, in 30% solution state {RER}|market for|Cut-off, U	Ecoinvent 3.6
Sodium Hydroxide seeds	Proxy datum: Sodium hydroxide, without water, in 50% solution state {GLO}|market for|Cut-off, U	Ecoinvent 3.6
Washing water	Tap water {RER}|market group for|Cut-off, U	Ecoinvent 3.6
Cooling water	Tap water {RER}|market group for|Cut-off, U	Ecoinvent 3.6
Electricity	Electricity, low voltage {IT}|market for|Cut-off, U	Ecoinvent 3.6
Transport	Transport, freight, lorry, unspecified {RER}|transport, freight, lorry, all sizes, EURO6 to generic market for|Cut-off, U	Ecoinvent 3.6
Wastewater treatment	Wastewater, average {Europe without Switzerland}|market for wastewater, average|Cut-off, U	Ecoinvent 3.6

## Supplementary Materials

The following are available online: Table S1: Normalisation and weighing factors to be used with the EF 3.0 impact assessment method. Table S2: Life cycle impact assessment of the six CNFs produced at the lab scale; the results are given per FU and refer to the normalisation stage (pure quantities). Table S3: Life cycle impact assessment of the six CNFs produced at the lab scale; the results are given per FU and refer to the single-score stage in arbitrary eco-points (Pt) × 10^-6^. Table S4: Life cycle impact assessment of the six CNFs produced at the lab scale; the results are given per FU and refer to the characterisation stage; scenario (2): reduction of the electricity consumption by 50% with respect to the base scenario (1). Table S5: Life cycle impact assessment of the six CNFs produced at the lab scale; the results are given per FU and refer to the characterisation stage; scenario (3): reduction of the electricity consumption by 75% with respect to the base scenario (1).

## Sample Availability

Not available.
