# Supplementary material for: Comparative Life Cycle Assessment of Cellulose Nanofibres Production Routes from Virgin and Recycled Raw Materials
Source: Molecules. 2021 Apr 28;26(9):2558. doi: 10.3390/molecules26092558 (PMC8125706; doi:10.3390/molecules26092558)
Supplement: Supplementary file 1 [file molecules-26-02558-s001.zip › molecules-1184637-Supplementary.pdf]

Supplementary Materials for

"Comparative Life Cycle Assessment of Cellulose Nanofibres Production Routes from Virgin and Recycled Raw Materials"

Table S1. Normalization and weighting factors to be used with EF 3.0 impact assessment method [1].

Impact category	Normalization	Weighting
Climate change	0.0001235	0.2106
Ozone depletion	18.64	0.0631
Ionising radiation	0.000237	0.0501
Photochemical ozone formation	0.02463	0.0478
Particulate matter	1680	0.0896
Human toxicity, non–cancer	4354	0.0184
Human toxicity, cancer	59173	0.0213
Acidification	0.018	0.062
Eutrophication, freshwater	0.6223	0.028
Eutrophication, marine	0.05116	0.0296
Eutrophication, terrestrial	0.005658	0.0371
Ecotoxicity, freshwater	0.00002343	0.0192
Land use	0.00000122	0.0794
Water use	0.00008719	0.0851
Resource use, fossils	0.00001538	0.0832
Resource use, minerals and metals	15.71	0.0755

Table S2. Life cycle impact assessment of the six CNFs produced at the lab scale; results are given per FU and refers to the normalization stage (pure quantities).

Impact Category	Unit	Enzymatic treatments & Homogenization (ENZHO)		TEMPO–Oxidation & Homogenization (TOHO)		TEMPO–Oxidation & Ultrasonication (TOSO)	
		Virgin fibres (hardwood kraft pulp)	Recycled materials (industrial waste sludge)	Virgin fibres (Cotton linters)	Recycled materials (industrial waste sludge)	Virgin fibres (Cotton linters)	Recycled materials (industrial waste sludge)
Climate change	–	2.29^{-5}	2.19×10^{-5}	7.81×10^{-5}	8.37×10^{-5}	1.09×10^{-4}	9.68×10^{-5}
Ozone depletion	–	4.74×10^{-7}	4.48×10^{-7}	1.53×10^{-6}	1.72×10^{-6}	2.16×10^{-6}	2.00×10^{-6}
Ionising radiation	–	5.48×10^{-6}	5.19×10^{-6}	1.72×10^{-5}	1.96×10^{-5}	2.41×10^{-5}	2.26×10^{-5}
Photochemical ozone formation	–	1.11×10^{-5}	1.00×10^{-5}	3.83×10^{-5}	3.84×10^{-5}	5.28×10^{-5}	4.43×10^{-5}
Particulate matter	–	8.61×10^{-6}	6.49×10^{-6}	3.21×10^{-5}	2.46×10^{-5}	4.29×10^{-5}	2.83×10^{-5}
Human toxicity, non–cancer	–	8.42×10^{-6}	7.79×10^{-6}	2.97×10^{-5}	2.90×10^{-5}	4.50×10^{-5}	3.30×10^{-5}
Human toxicity, cancer	–	4.33×10^{-6}	3.79×10^{-6}	2.35×10^{-5}	1.45×10^{-5}	3.04×10^{-5}	1.64×10^{-5}
Acidification	–	1.83×10^{-5}	1.72×10^{-5}	7.43×10^{-5}	6.57×10^{-5}	1.01×10^{-4}	7.59×10^{-5}
Eutrophication, freshwater	–	3.37×10^{-5}	3.19×10^{-5}	1.31×10^{-4}	1.21×10^{-4}	1.78×10^{-4}	1.39×10^{-4}
Eutrophication, marine	–	8.13×10^{-6}	7.28×10^{-6}	1.08×10^{-4}	2.86×10^{-5}	1.36×10^{-4}	3.31×10^{-5}
Eutrophication, terrestrial	–	1.10×10^{-5}	9.99×10^{-6}	5.66×10^{-5}	3.82×10^{-5}	7.51×10^{-5}	4.42×10^{-5}
Ecotoxicity, freshwater	–	1.03×10^{-4}	9.35×10^{-5}	3.15×10^{-4}	2.64×10^{-4}	4.16×10^{-4}	3.07×10^{-4}
Land use	–	4.29×10^{-6}	1.25×10^{-6}	9.56×10^{-6}	6.64×10^{-6}	1.24×10^{-5}	7.74×10^{-6}
Water use	–	3.39×10^{-5}	3.18×10^{-5}	1.89×10^{-4}	6.74×10^{-5}	2.04×10^{-4}	4.67×10^{-5}
Resource use, fossils	–	4.16×10^{-5}	3.96×10^{-5}	1.36×10^{-4}	1.52×10^{-4}	1.90×10^{-4}	1.75×10^{-4}
Resource use, minerals and metals	–	2.47×10^{-5}	2.23×10^{-5}	8.42×10^{-5}	8.47×10^{-5}	1.16×10^{-4}	9.73×10^{-5}

Table S3. Life cycle impact assessment of the six CNFs produced at the lab scale; results are given per FU and refers to the single score stage in arbitrary eco−points (Pt) × 10^{-6}.

Impact Category	Unit	Enzymatic treatments & Homogenization (ENZHO)		TEMPO−Oxidation & Homogenization (TOHO)		TEMPO−Oxidation & Ultrasonication (TOSO)	
		Virgin fibres (hardwood kraft pulp)	Recycled materials (industrial waste sludge)	Virgin fibres (Cotton linters)	Recycled materials (industrial waste sludge)	Virgin fibres (Cotton linters)	Recycled materials (industrial waste sludge)
Total	µPt	$1.99\times10^{+1}$	$1.83\times10^{+1}$	$7.73\times10^{+1}$	$6.37\times10^{+1}$	$1.01\times10^{+2}$	$7.09\times10^{+1}$
Climate change	µPt	4.83×10	4.61×10	$1.64\times10^{+1}$	$1.76\times10^{+1}$	$2.29\times10^{+1}$	$2.04\times10^{+1}$
Ozone depletion	µPt	2.99×10^{-2}	2.83×10^{-2}	9.66×10^{-2}	1.09×10^{-1}	1.36×10^{-1}	1.26×10^{-1}
Ionising radiation	µPt	2.75×10^{-1}	2.60×10^{-1}	8.64×10^{-1}	9.84×10^{-1}	1.21×10	1.13×10
Photochemical ozone formation	µPt	5.33×10^{-1}	4.80×10^{-1}	1.83×10	1.83×10	2.53×10	2.12×10
Particulate matter	µPt	7.72×10^{-1}	5.81×10^{-1}	2.88×10	2.20×10	3.85×10	2.54×10
Human toxicity, non−cancer	µPt	1.55×10^{-1}	1.43×10^{-1}	5.46×10^{-1}	5.34×10^{-1}	7.46×10^{-1}	6.07×10^{-1}
Human toxicity, cancer	µPt	9.22×10^{-2}	8.07×10^{-2}	5.01×10^{-1}	3.09×10^{-1}	6.47×10^{-1}	3.50×10^{-1}
Acidification	µPt	1.14×10	1.07×10	4.61×10	4.07×10	6.27×10	4.71×10
Eutrophication, freshwater	µPt	9.45×10^{-1}	8.92×10^{-1}	3.67×10	3.39×10	4.99×10	3.90×10
Eutrophication, marine	µPt	2.41×10^{-1}	2.15×10^{-1}	3.20×10	8.46×10^{-1}	4.01×10	9.80×10^{-1}
Eutrophication, terrestrial	µPt	4.07×10^{-1}	3.71×10^{-1}	2.10×10	1.42×10	2.79×10	1.64×10
Ecotoxicity, freshwater	µPt	1.98×10	1.79×10	6.05×10	5.06×10	8.00×10	5.89×10
Land use	µPt	3.41×10^{-1}	9.95×10^{-2}	7.59×10^{-1}	5.27×10^{-1}	9.85×10^{-1}	6.14×10^{-1}
Water use	µPt	2.88×10	2.71×10	$1.61\times10^{+1}$	5.74×10	$1.73\times10^{+1}$	3.98×10
Resource use, fossils	µPt	3.46×10	3.30×10	$1.13\times10^{+1}$	$1.26\times10^{+1}$	$1.58\times10^{+1}$	$1.46\times10^{+1}$
Resource use, minerals and metals	µPt	1.87×10	1.69×10	6.36×10	6.40×10	8.74×10	7.35×10

Table S4. Life cycle impact assessment of the six CNFs produced at the lab scale; results are given per FU and refers to the characterization stage; scenario (2): reduction of electricity consumption by 50% with respect to base scenario (1).

Impact Category	Unit	Enzymatic treatments & Homogenization (ENZHO)		TEMPO–Oxidation & Homogenization (TOHO)		TEMPO–Oxidation & Ultrasonication (TOSO)	
		Virgin fibres (hardwood kraft pulp)	Recycled materials (industrial waste sludge)	Virgin fibres (Cotton linters)	Recycled materials (industrial waste sludge)	Virgin fibres (Cotton linters)	Recycled materials (industrial waste sludge)
Climate change	kg CO2 eq	9.65×10^{-2}	9.00×10^{-2}	3.44×10^{-1}	3.44×10^{-1}	4.74×10^{-1}	8.24×10^{-1}
Ozone depletion	kg CFC11 eq	1.33×10^{-8}	1.23×10^{-8}	4.32×10^{-8}	4.74×10^{-8}	6.11×10^{-8}	1.14×10^{-7}
Ionising radiation	kBq U–235 eq	1.23×10^{-2}	1.13×10^{-2}	3.79×10^{-2}	4.24×10^{-2}	5.23×10^{-2}	1.00×10^{-1}
Photochemical ozone formation	kg NMVOC eq	2.49×10^{-4}	2.09×10^{-4}	8.98×10^{-4}	7.96×10^{-4}	1.22×10^{-3}	1.90×10^{-3}
Particulate matter	disease inc.	3.24×10^{-9}	2.02×10^{-9}	1.30×10^{-8}	7.59×10^{-9}	1.70×10^{-8}	1.80×10^{-8}
Human toxicity, non–cancer	CTUh	1.11×10^{-9}	9.86×10–10	4.17×10^{-9}	3.59×10^{-9}	5.57×10–9	8.43×10^{-9}
Human toxicity, cancer	CTUh	4.41×10^{-11}	3.56×10^{-11}	3.04×10^{-10}	1.37×10^{-10}	3.81×10^{-10}	3.07×10^{-10}
Acidification	mol H+ eq	5.39×10^{-4}	4.87×10^{-4}	2.58×10^{-3}	1.85×10^{-3}	3.44×10^{-3}	4.44×10^{-3}
Eutrophication, freshwater	kg P eq	2.91×10^{-5}	2.66×10^{-5}	1.29×10^{-4}	1.00×10^{-4}	1.72×10^{-4}	2.37×10^{-4}
Eutrophication, marine	kg N eq	9.21×10^{-5}	7.71×10^{-5}	1.90×10^{-3}	3.09×10^{-4}	2.35×10^{-3}	7.24×10^{-4}
Eutrophication, terrestrial	mol N eq	1.05×10^{-3}	9.00×10^{-4}	7.15×10^{-3}	3.44×10^{-3}	9.24×10^{-3}	8.23×10^{-3}
Ecotoxicity, freshwater	CTUe	3.25×10	2.87×10	9.74×10	6.96×10	$1.26\times10^{+1}$	$1.53\times10^{+1}$
Land use	Pt	3.00×10	5.23×10^{-1}	6.17×10	3.51×10	7.82×10	7.96×10
Water use	m3 depriv.	3.37×10^{-1}	3.15×10^{-1}	2.00×10	5.81×10^{-1}	2.10×10	5.66×10^{-1}
Resource use, fossils	MJ	1.41×10	1.31×10	4.66×10	5.01×10	6.46×10	$1.20\times10^{+1}$
Resource use, minerals and metals	kg Sb eq	8.85×10^{-7}	7.50×10^{-7}	3.14×10^{-6}	2.82×10^{-6}	4.24×10^{-6}	6.64×10^{-6}

Table S5. Life cycle impact assessment of the six CNFs produced at the lab scale; results are given per FU and refers to the characterization stage; scenario (3): reduction of electricity consumption by 75% with respect to base scenario (1).

**Impact Category**	**Unit**	**Enzymatic treatments & Homogenization (ENZHO)**		**TEMPO–Oxidation & Homogenization (TOHO)**		**TEMPO–Oxidation & Ultrasonication (TOSO)**	
		Virgin fibres (hardwood kraft pulp)	Recycled materials (industrial waste sludge)	Virgin fibres (Cotton linters)	Recycled materials (industrial waste sludge)	Virgin fibres (Cotton linters)	Recycled materials (industrial waste sludge)
Climate change	kg CO2 eq	5.18×10^{-2}	4.64×10^{-2}	2.01×10^{-1}	1.77×10^{-1}	2.71×10^{-1}	2.03×10^{-1}
Ozone depletion	kg CFC11 eq	7.32×10^{-9}	6.38×10^{-9}	2.38×10^{-8}	2.48×10^{-8}	3.37×10^{-8}	2.91×10^{-8}
Ionising radiation	kBq U–235 eq	6.90×10^{-3}	6.05×10^{-3}	2.05×10^{-2}	2.21×10^{-2}	2.76×10^{-2}	2.48×10^{-2}
Photochemical ozone formation	kg NMVOC eq	1.47×10^{-4}	1.09×10^{-4}	5.70×10^{-4}	4.15×10^{-4}	7.53×10^{-4}	4.75×10^{-4}
Particulate matter	disease inc.	2.30×10^{-9}	1.10×10^{-9}	1.00×10^{-8}	4.06×10^{-9}	1.27×10^{-8}	4.61×10^{-9}
Human toxicity, non–cancer	CTUh	7.00×10^{-10}	5.84×10^{-10}	2.84×10^{-9}	2.05×10^{-9}	3.70×10^{-9}	2.23×10^{-9}
Human toxicity, cancer	CTUh	2.95×10^{-11}	2.14×10^{-11}	2.57×10^{-10}	8.22×10^{-11}	3.15×10^{-10}	8.84×10^{-11}
Acidification	mol H+ eq	2.99×10^{-4}	2.53×10^{-4}	1.81×10^{-3}	9.58×10^{-4}	2.34×10^{-3}	1.10×10^{-3}
Eutrophication, freshwater	kg P eq	1.65×10^{-5}	1.44×10^{-5}	8.88×10^{-5}	5.32×10^{-5}	1.15×10^{-4}	6.01×10^{-5}
Eutrophication, marine	kg N eq	5.87×10^{-5}	4.45×10^{-5}	1.79×10^{-3}	$1.84\times10_{-4}$	2.19×10^{-3}	2.13×10^{-4}
Eutrophication, terrestrial	mol N eq	6.06×10^{-4}	4.68×10^{-4}	5.72×10^{-3}	1.78×10^{-3}	7.22×10^{-3}	2.04×10^{-3}
Ecotoxicity, freshwater	CTUe	2.68×10	2.31×10	7.89×10	4.81×10	9.94×10	5.63×10
Land use	Pt	2.74×10	2.71×10^{-1}	5.34×10	2.54×10	6.64×10	2.98×10
Water use	m3 depriv.	3.11×10^{-1}	2.89×10^{-1}	1.92×10	4.84×10^{-1}	1.98×10	2.02×10^{-1}
Resource use, fossils	MJ	$7.60\times10_{-1}$	6.78×10^{-1}	2.57×10	2.59×10	3.51×10	2.96×10
Resource use, minerals and metals	kg Sb eq	$5.41\times10_{-7}$	4.15×10^{-7}	2.04×10^{-6}	1.53×10^{-6}	2.67×10^{-6}	1.72×10^{-6}

References

1. Fazio, S.; Castellani, V.; Sala, S.; Schau, E.M.; Secchi, M.; Zampori, L.; Diaconu, E. *Supporting information to the characterisation factors of recommended EF Life Cycle Impact Assessment methods*; 2018; ISBN 9789279767425.
